# A Psychological Perspective on Preterm Children: The Influence of Contextual Factors on Quality of Family Interactions

**DOI:** 10.1155/2017/9152627

**Published:** 2017-10-12

**Authors:** Michela Gatta, Marina Miscioscia, Lorenza Svanellini, Chiara Peraro, Alessandra Simonelli

**Affiliations:** ^1^Childhood Adolescence Family Unit, ULSS6 Veneto, Padua, Italy; ^2^Department of Women's and Children's Health, Padua University, Padua, Italy; ^3^Department of Developmental and Social Psychology, Padua University, Padua, Italy

## Abstract

Preterm birth has a critical influence on interactive, communicative, and expressive child behaviour, particularly during the first years of life. Few studies have stressed the assessment of mother-father-child interaction in families with preterm children, generating contradictory results. The present study wished to develop these fields: (i) comparing the quality of family interactions between families with preterm children and families with children born at full term; (ii) observing the development of family interactions after six months in the families with children born preterm; (iii) assessing family and contextual factors, as parental stress and social support, in parents of preterm children in order to observe their influence on the quality of family interactions. 78 families are recruited: 39 families with preterm children (*M* = 19,8 months, SD = 11,05) and 39 families with full-term children (*M* = 19,66 months; SD = 13,10). Results show that families with preterm children display a low quality of mother-father-child interactions. After six months, family interactions result is generally stable, except for some LTP-scales reflecting a hard adjustment of parenting style to the evolution of the child. In families with preterm children, the parenting stress seemed to be correlated with the quality of mother-father-child interactions.

## 1. Introduction

Every year, in the world, an estimated 15 million babies are born preterm and prematurity is considered the leading cause of neonatal mortality and the second cause of death before 5 years of age [1]. Among them, about 5% of these children are born before 28 weeks, 15% between 28 and 31 weeks, and about 20% between 32 and 33; finally, between 60 and 70% of them are born between 34 and 36 weeks' gestation [2]. Across all Europe the survival of preterm children, born before 37 weeks of gestational age (GA), has recently increased, reaching an incidence of 7–10% [3]. In Italy, the prevalence rate of premature birth is about 6,5% [4].

Regarding the children's behavioural and emotional development, preterm infants show weak relational, emotional, and social skills and difficulties in self-regulation already in the early stages of their development [5]. Specifically, most studies show the presence of higher mean scores on socioemotional scales in preterm children, than their peers born at term, even not reaching clinical cut-off [6].

Several studies that have explored the development of preterm child have shown that poor social/interactive skills, poor behavioural and emotional self-regulation, emotional difficulties, and reduced attention are the most common behaviour problems in preterm infants and children [5].

Currently, literature is also focusing on parental distress. Parents undergo great suffering and concern regarding child's health, describing a stressful and emotional experience related to parenting. Specifically, some researchers were focused on parenting stress, assessed with the Parenting Stress Index-Short Form [7]. In Gray and colleagues [8] study with mothers of children aged 12 months (corrected age) those whose offspring were born preterm experienced twice as much stress as the mothers of those born at term. The main difference emerged on the parent-infant dysfunctional interaction scale assessed with the Parent-Child Early Relational Assessment [9, 10]. This result suggests that, in the first year of child's life, mothers of preterm babies had more difficulty connecting with their child, with respect to the mothers of at-term children [8]. Considering the parental stress characteristics of parents of preterm children, some studies have noticed the level of perceived social support, as it could be a protective factor for both parents' well-being and mental health since it could be able to reduce parents' stress [11–13]. Actually, the means of perceived social support of “preterm parents” are 5 points higher than the ones perceived from “at-term parents,” underscoring its importance in the case of high level of stress [12]. Singer and colleagues [13] also found that the extremely stressful conditions relating to preterm birth do not reduce the adults' perception of their parenting competencies.

Regarding the contribution of the child to the interactions, preterm children are seen as being more passive [14–16], less attentive, and less concentrated and responsive [16–20]. Preterm children appeared to be less inclined to make eye contact with their primary caregiver [21–23] and may be less vocal [20, 24] or more vocal [25] but with less contingency [18]. With respect to children born at term, the preterm ones have less well-developed autoregulatory competencies [26] and little smiles [27] and are mostly described by the expression of more negative emotions [16, 21, 28, 29]. Finally, the preterm children also find it more difficult to give clear clues to caregivers [30, 31].

Concerning maternal interactive style, studies find that the mothers of preterm children are more directive, active, and controlling at 3 months [14, 17, 24, 25] and tend to be less sensitive [14, 24], using a directive scaffolding [32, 33] with a contradictory style, which alternates between passive and overstimulating moments [34]. In summary, many researchers have identified a specific interactive style in the preterm mother-child dyad, in which the child seems to be more passive in his/her interaction with the mother. Some authors attribute this characteristic to a maternal intrusiveness, while others hold that it is related to the maternal reactivity to compensate the child's developmental inadequacy [23, 35].

Regarding affection, while some studies that involved a group of heterogeneous preterm children found no differences [26, 28] others found that mothers of children born extremely preterm mainly express neutral emotions [36]. Some authors found that some characteristics of interaction with preterm children become progressively richer after the first six months of child's life when the environment becomes more complex and demanding [19, 28, 32, 37]. In this regard, a research of Feldman and Eidelman [30] has highlighted that mother's postpartum interactive style predicts both maternal and paternal interactive synchrony with their child during his development.

One of the critical factors of these researchers is that these studies have generated ambiguities due to the way to assess the adult-child interactions and to the heterogeneity of the samples considered [35, 38, 39]. In this lack of knowledge, only a few studies have addressed the assessment of triadic interactions in families with preterm children [36, 40, 41]. In a recent study on the individual, dyadic, and triadic influences on the development of the family system, Feldman [42] found that the infant-risk families (composed of families with preterm children and families with children affected by intrauterine growth restriction) displayed the lowest cohesion and highest rigidity, compared to four group of families: controls and three mother-risk groups (depressed, anxious, and comorbid). Only a recent study used the Lausanne Trilogue Play [43], an observational method also used in the research described in this paper. Only a few variables (regarding affect sharing, timing/synchronization, and child behaviour) were used to observe the quality of family interactions in 83 families with 6-month-old healthy children born between the 28th and the 34th week of gestation, and no differences emerged from the comparison with the control group. This study was the first to use the LTP approach to assess this construct.

In this line, our previous study [44] has attempted to compare the quality of family interactions in a group of families with preterm children and a group of families with children born at full term, exploring differences and similarities. Results show differences in the quality of family interactions emerged between the preterm and at-term children groups. The preterm group showed a significantly lower quality of family interactions than the at-term group. Another aim of this prior research was to consider the associations between the quality of family interactions and contextual factors, as parental empowerment, child's temperament, parental stress, and perceived social support. The parental stress of both parents related to their parental empowerment and maternal stress was related, also, to the partner's parental empowerment. Social support had a positive influence on parental stress, with maternal stress also related to the perceived social support from the partner, which underscores the protective role of the father on the dyad [44].

This paper aims to develop our previous study that contributed to literature regarding family interaction in families with preterm children. The first aim of the present study is to increase the previous sample with a higher size of families with preterm children, comparing the results with those obtained in a group of families with at-term children. We expected to confirm the results of our previous study, which is to find a significant difference between the two observed groups.

Secondly, we aim to observe the evolution of family interactions after six months, retesting a small group of families who have accepted to return for a follow-up.

Finally, we aim to observe if contextual and family variables, as perceived parental stress and social support are influencing factors on the quality of the family interactions. In detail, we expect to observe a significant influence of these aspects on the couple's degree of supportive cooperation during the interactions with the child and on their parental competence to interact with him, eliciting his involvement in the joined activity.

## 2. Methods

### 2.1. Participants and Procedures

78 children and their families are recruited for this research project. The preterm group included 39 families and their children (*M* = 19,8 months, SD = 11,05). Families are recruited from two different Italian organizations that offer support and intervention for preterm children and their families: a private Onlus Association “Il Pulcino” (with a recruitment of 31 children) and the Neurorehabilitation Service part of the Children, Adolescents and Families Unit of the Public Health Service ULSS6 in Padua (with a recruitment of 8 children).

Families attending the Neurorehabilitation Service are recruited by a child neuropsychiatrist, who explained the purpose of the research. Families who are part of the “Il Pulcino” Onlus Association are recruited by the professionals of the association, who explained the purpose of the study and, depending on the families' consent, placed them in contact with the responsible research project. All the parents taking part in the project gave and signed their informed consent to the study, approved by the Ethical Committee (CEP 204 SC).  Table 1 shows the characteristics of the preterm children and families group.

Mothers have a mean of 38,13 years of age (SD = 4,16) and fathers of 41,4 years (SD = 5,37). The 51,3% of mothers and the 46,2% of fathers have achieved a secondary school degree.

14 families, among those, have been invited to participate in a follow-up section, after six months. In this subgroup children have a mean of 21,5 months of correct age (SD = 12,1); 8 of them are male and 6 are female.  Table 2 shows the characteristics of this subgroup.

The control group employed in this study is part of a longitudinal study regarding the development of family interactions [45]. This project involved a hundred of couples who spontaneously conceived their first child, who were followed up from the 7th month of pregnancy until their child was 48 months old. A group of 39 children (*M* = 19,66 months; SD = 13,10) and their families was drawn from this sample, to match the preterm group in terms of the child's age and gender and the parents' ages. The parents have a mean of 36,74 years of age (SD = 3,85).

The Lausanne Trilogue Play Procedure [43] is administered to both groups of families to assess the quality of their family interactions. The following questionnaires are also administered to the group with preterm children: the Parenting Stress Index-Short Form [7] and the Multidimensional Scale of Perceived Social Support [46]. The observational procedure and the questionnaires are administered at the Childhood, Adolescence and Family Unit (ULSS6 of Padua) to all the families of the preterm group.

## 3. Materials

*Lausanne Trilogue Play [43]* is a semistandardized observation situation designed to assess the quality of family interactions. The administration involved the mother-father-child triad–invited to cooperate and work together in order to conduct an activity. The proposed activity is a play session or the planning of a birthday party or a family trip, in connection with child's age. Detailed instructions invited the family to organize the activity, as they usually do at home, just following four rules which reflect these four relational configurations: (I) at first, just one parent interacted with his child, while the other one stayed simply present; (II) then, parents reverse the roles, so that the one who was simply present became the active partner, and vice versa; (III) parents and child play all together; (IV) parents interacted while the child stay simply present. The session was videotaped and later scored using the Family Alliance Assessment Scale (FAAS) 6.3 [47] composed of 15 observational variables (*the variables are grouped into macrocategories: participation (postures and gazes, inclusion of partners), organization (role implication, structure), focalization (parental scaffolding, coconstruction), affect sharing (family warmth, validation, and authenticity), timing/synchronization (interactive mistakes during activities, interactive mistakes during transitions), coparenting (support, conflicts), and infant (involvement, self-regulation)*[48, pp. 24]).

*Parenting Stress Index-Short Form (PSI-SF [7])* is a self-report questionnaire that aims to identify stressful parent-child relational systems at risk of leading to dysfunctional behaviour on the part of the parent or the child. The short form (the only one validated in Italy) comprises 36 items scored on a Likert scale from 1 (strongly agree) to 5 (strongly disagree). The items are divided into three scales: (1) parental stress that assesses parent's feelings of being trapped in the parenting role; (2) parent-child dysfunctional interaction that measures the nature of the interaction between parent and child; (3) difficult child that assesses parents' perceptions of their children. Scores above the 85th percentile on the total stress scale are considered borderline clinically significant [49].

*Multidimensional Scale of Perceived Social Support (MSPSS [46], Italian version [50])* is a brief self-report scale composed of 12 items that measure three areas: perceived social support from family, from friends, and from significant others. Answers can be scored on a Likert scale from 1 (strongly agree) to 7 (strongly disagree). The instrument has no cut-off score. The score range is from 84 (maximum) to 12 (minimum).

## 4. Results

### 4.1. Preliminary Analysis

As shown in Table 1, the group of preterm children is composed of children with different gestational age, and it includes children with disabilities. A one-way ANOVA has been carried out in order to detect differences, due to the degree of prematurity (gestational age) and to the presence of child disability, in the different variables investigated by the applied tools (parental stress, social support, and quality of family interactions). The ANOVA, confirmed by Bonferroni's post hoc test, does not underline differences between groups about parental stress (PSI) for both degrees of prematurity (mothers total stress: *F*(2,36) = 1.598; *p* = 0.216; fathers total stress: *F*(2,36) = 2.392; *p* = 0.106) and the presence of child disability (mother total stress: *F*(1,37) = 2.280; *p* = 0.140; father total stress: *F*(1,37) = 2.077; *p* = 0.158).

**Figure 1:**
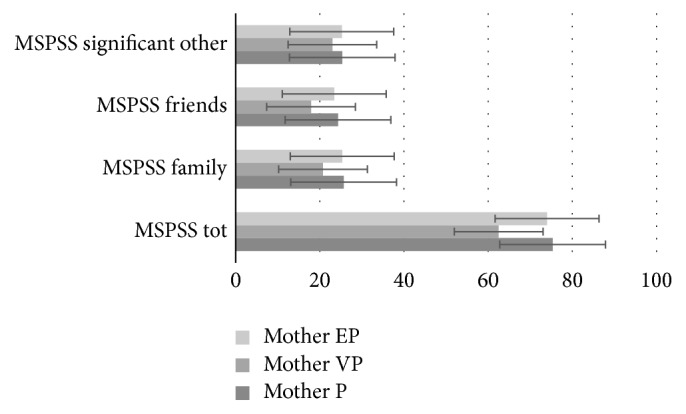
Means of MSPSS scores of each preterm subgroups. ^*∗*^*Note*. MSPSS: Multidimensional Scale of Perceived Social Support; EP: extremely preterm; VP: very preterm; P: preterm.

Some significant differences linked to the degree of prematurity emerge for mothers, on the variables of the Multidimensional Scale of Perceived Social Support (Figure 1). There are statistically significant differences in the perceived support total score, *F*(2,30) = 8.151, *p* = 0.001, family support, *F*(2,30) = 3.99, *p* = 0.029, and friends support, *F*(2,30) = 9.54, *p* = 0.01. Bonferroni's post hoc test shows that mothers of very preterm children perceived low social support compared to mothers of low preterm children; these differences have been detected in the total score (*p* = 0.002) and in the family support scale (*p* = 0.045) and in the friends support scale (*p* = 0.01). No difference was observed, instead, for child disability, *F*(1,31) = .808 and *p* = 0.376.

Once more, no differences emerge for fathers in the perceived support total score for both children's groups based on the degrees of prematurity, *F*(1,30) = 1.679 and *p* = 0.204, and on the presence of child disability: *F*(1,31) = 2.307; *p* = 0.139.

Regarding the quality of family interactions, no differences linked to the degree of prematurity emerged from the ANOVA in the LTP total score *F*(2,36) = 3.023; *p* = 0.061; and furthermore none linked to the presence of child disability *F*(1,37) = 0.009; *p* = 0.926. As a result of these preliminary analyses, the preterm group was judged to be homogenous.

### 4.2. Family Interactions

Our first aim was to compare the quality of family interactions in two groups of families: families with preterm children and families with children born at full term.

**Figure 2:**
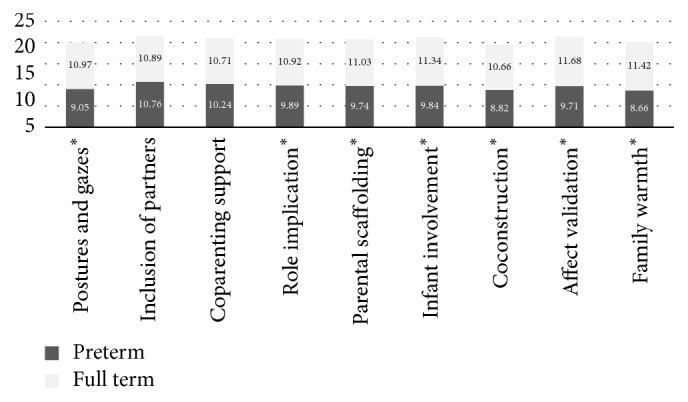
Means of LTP variable scores of each studied group. ^*∗*^*p* < 0.05.

Having confirmed the homogeneity of the preterm group (degree of prematurity and child disabilities), a *t*-test was run to compare the quality of the family interactions between the preterm group and the full-term group, whose results are given in Table 3.  Figure 2 shows the means of the compared LTP variables (LTP of the control group has been coded with the FAAS 4.0 that has 10 variables; 9 of them match with 9 variables of the FAAS 6.3 coding system; the comparison has been done on these nine variables and on the sum of the LTP parts); between them, only two variables do not have different results: the variable inclusion of the partner, *t*(74) = −.400 and *p* = 0.690, and coparenting support, *t*(74) = −1.393 and *p* = 0.168.

### 4.3. Development of Family Interactions

The second aim of the present study was to observe the development of family interactions in the group of families with preterm children. Six months after the first observation, 14 families have participated in a follow-up session. Wilcoxon test shows a significant change in four of the LTP variables; looking at the means, three of them show a significant decrease: scaffolding (*Z* = −2.326; *p* = 0.020), interactive mistakes during the transitions (*Z* = −2.473; *p* = 0.013), and authenticity (*Z* = −2.38; *p* = 0.017). A significant increase is observed in the means of the scoring of the family warmth variable (*Z* = −2.335; *p* = 0.020).

### 4.4. Factors of Influence

The third aim of the present study was to observe if contextual and family variables, as perceived parental stress and social support, are influencing factors on the quality of the family interactions. Before verifying the association, we wondered if mothers and fathers PSI scores correlated between them and if they reported different levels of parental stress.

Table 4 shows the correlations between mother and father PSI scores.

A Paired-Sample *T*-test between mother's and father's parental stress total score confirms that no significant difference has been found among them: *t*(38) = −.620, *p* = 0.539. Also regarding the perception of social support, mother's and father's scores show a significant correlation (*r* = .511; *p* = 0.002). Once again, a Paired-Sample *T*-test between mother's and father's perceived support total score affirms no difference between them: *t*(32) = −.598, *p* = 0.554.

Following these results, we have performed Pearson's correlations between PSI, MSPSS, and LTP.  Table 5 shows the significant results.

No significant results emerge from the perceived social support scales and family interactions.

## 5. Discussion

Triadic interactive dynamics in families with preterm children have been very little investigated. A small body of literature has not shown significant differences in the comparison with families with children born at full term [51]. Our study fits in this direction, trying to understand the quality of the family interactions and their evolution in the time. From the comparison it has emerged that the group of families with at-term children shows a great quality of triadic interactions, as is deduced by more elevated scores in almost all the LTP variables. The only variables where differences are not revealed are “inclusion” and “coparenting support”; these two variables underline a good mutual support in the parental couple. According to a recent study of Adama and colleagues [52], couples that have faced a premature delivery perceive the partner's support as more significant. In our study, apart from the positive correlations observed, also between mothers and fathers significant agreement emerges regarding the perception of social support and parental stress and from the absence of differences among their PSI scores. The specific differences encountered in the Lausanne Trilogue Play scales, between preterm group and control group, show specific difficulties in the triadic interactions in the preterm sample. Due to the mentioned risk factors involving parents and preterm children, several studies have focused on parent-child interactions with the aim of investigating the general quality of the early adult-child relationship and development. Globally, data show that prematurity has a negative influence on interactive, communicative, and expressive levels of mother-child interaction during the first years of life [35, 38]. Our results confirm these pieces of evidence also at the triadic level (mother-father-child interactions).

Regarding the development of the family interactions, they generally have stable results after six months which are considered, even if we notice some significant variation: the triadic family interactions seem in fact to worsen after six months in the dimensions of scaffolding, authenticity, and interactive errors, while they are improving in the dimension of the family warmth.

Preterm children seem to demonstrate, in the first years of life, a smaller level of vigilance, attention, activity, and responsivity compared to children born at full term [27]; this difference seems to be reduced with their growth [16].

Similarly, even if parent behaviour shows an elevated stability, according to the literature it seems to become mainly intrusive [16]. The mean age of our sample at the time of the first observation was of 21,5 months and of 28,2 months in the follow-up.

During the second observation, children of preterm sample have participated more actively in the game, as observed by the scores obtained in the variable child's involvement. This might contribute to mainly activating some interactive errors and make the adaptation harder for the parents to the real abilities of their child through scaffolding. It seems therefore that parents fail to evolve, in this range of time, their ability to involve their child in an interactive exchange, probably anchored to a vision of a child as always small, immature, and not very active, as observed in the literature [53].

The atypical growth that characterizes the development of premature children could explain the difficulty of parents to adapt positively to the child's acquisitions. In fact, after birth, premature babies undergo to rapid “catch-up” [54], rapid and sudden acquisitions of skills that may not allow the parent to adapt in a functional way. At the same time, however, the greater involvement of the child in the gameplay is also associated with greater family warmth, as if it would be easier for the parents to catch the affective states of the child and respond appropriately. Some studies found that parents of preterm infants are sensitive and responsive in the interaction [22, 28, 55] but they tend to express responsiveness verbally more than in their facial expressions [25]. They use social monitoring and eye contact [39] and positive affection expressed verbally and nonverbally [26], although birth weight influences the intrusiveness of mothers [39].

The lower authenticity could be influenced by the repetition of the second observational procedure.

Another aim of our study was to observe the influence of parental stress and perception of social support experienced by these parents, on the quality of family interaction. In our preterm group the mean of the parental stress total score does not exceed the clinical cut-off; but, in agreement with the literature [56], it seemed to be higher. Moreover, literature also means that mothers reported greater stress than fathers did, and these differences remained remarkably stable over time [57]. This gender difference was not confirmed in our study and it brings hypothesis that these children are exposed, consistently, to the distressed parental cares. This relationship could affect child symptomatology [58].

Perceived stress is associated with different variables of the Lausanne Trilogue Play and we can see that the subscales of maternal stress are mainly associated with components of the triadic interactions concerning parental and family interaction (support and cooperation, partner inclusion, and coconstruction), while the paternal stress is more associated with aspects of interactions related to the child (self-regulation). In this line, Olafsen and colleagues [59] reached an association between parenting stress and negative reactive temperament in the child, at one year of child's life.

These are negative type correlations, thus when perceived stress increases, the LTP-scale score decreases. Paternal stress correlates positively with some of the LTP-scales, especially with the ability to solve interactive errors and to respect roles. This result could be explained by the tendency of fathers in paying more attention, during the interaction, to the game structure rather than to the relationship. Perception of less support may be associated with the condition of bigger vulnerability that children born at a lower gestational age can have, which often leads parents to limit contact with the outside world, and this could lead, in the first period, to an experience of social isolation [60].

## 6. Conclusions

The research presented expands, confirms, and is in continuity with the findings of the previous studies, highlighting the presence of some limits in triadic family interactions in families with preterm children. The families involved, in fact, obtain lower scores than the normative sample in seven of the nine compared LTP-scales. Therefore, such families show a good level of parenting support and cooperation and parental alliance, a strength of this group. They also show a level equal to the regulatory one in creating a good interactive environment through bodily signals.

After six months, this group of families seems to show a certain stability in the triadic interactions excluding the dimensions of scaffolding, authenticity, and solution of interactive errors in which they worsen, reflecting a hard adjustment of parenting style to the evolution of the child and therefore, generally, a difficult triadic adaptation.

This finding draws attention to family development in cases of prematurity of the child, to support the parental couple and the child along with its growth. It can occur that parents might cling to an idea of a child being immature and fragile, without being ready to change some interactive patterns in relation to growth.

Although compared to the two previous studies the sample has increased considerably, its sample size is not yet sufficient to generalize the results. It is, therefore, necessary to continue to involve new families in the different observations and above all to carry on a follow-up, in order to generalize the data and get more accurate information on the specificity of these families.

In order to offer them a support adjusted to their needs, current resources and the difficulties are listed.

This study opens several future prospects by observing its results; among these, there is the need to deepen the differences in triadic interactions and constructs analysed according to the degree of prematurity, particularly in the group of families with very preterm children and disability. It would also be interesting to continue medium and long-term observations, to investigate how the triadic interactions evolve over time, taking in count the second childhood as well. This kind of longitudinal design could underline the importance of adopting an intervention-research approach [61].

## Figures and Tables

**Table 1:** Preterm group description.

	Preterm full group		Preterm		Very preterm		Extremely preterm	
	*μ*	*σ*	*μ*	*σ*	*μ*	*σ*	*μ*	*σ*
Chronological age (months)	19.8	11.5	19.2	10.6	24.9	12.8	22.6	11.8
Corrected age (months)	19.5	11.3	18.2	10.5	22.5	12.7	19.5	11.3
Birth weight (g)	1294	602.1	1896	599	1121.7	227.65	818	246.19
Gestational age (weeks)	29.66	3.31	33.15	1.14	29.93	1.49	25.58	1.16

	*N* (%)		*N* (%)		*N* (%)		*N* (%)	

Disability	9 (23%)		3 (23%)		2 (14%)		4 (33%)	
Twins	9 (23%)		2 (15%)		4 (28,5%)		3 (12%)	
Male	24 (61,5%)		7 (53,8%)		8 (57,1%)		9 (75%)	
Female	15 (38,5%)		6 (46,2%)		6 (42,9%)		3 (25%)	
Preterm (P)	13 (33,3%)							
Very preterm (VP)	14 (35,9%)							
Extremely preterm (EP)	12 (30,8%)							

**Table 2:** Preterm group description at follow-up.

	Preterm full group		P		VP		EP	
	*μ*	*σ*	*μ*	*σ*	*μ*	*σ*	*μ*	*σ*
Chronological age (months)	23.7	12.1	24.5	13.09	28	16.49	18.25	2.98
Corrected age (months)	21.5	12.1	22.83	12.71	25.75	16.7	15.12	2.7
Birth weight (g)	1463.43	777.01	2135	707.7	1079	252.69	840	317
Gestational age (weeks)	30.57	3.08	33.1	1.47	30.75	1.35	26.5	0.57

	*N* (%)		*N* (%)		*N* (%)		*N* (%)	

Disability	5 (35,7%)		1 (16%)		1 (25%)		3 (75%)	
Twins	3 (21,4%)		0		3 (75%)		0	
Male	8 (57,1%)		4 (66,6%)		1 (25%)		3 (75%)	
Female	6 (42,9%)		2 (33,4%)		3 (75%)		1 (25%)	
Preterm (P)	4 (28,55%)							
Very preterm (VP)	4 (28,55%)							
Extremely preterm (EP)	6 (42,9%)							

**Table 3:** *T*-test between preterm group and control group.

	Preterm (*N* = 39)		At term (*N* = 39)		*t*	*p*
	*μ*	*σ*	*μ*	*σ*		
Part I	21.66	4.187	25.37	2.432	−4.724	0.000
Part II	22.42	3.492	25.58	2.585	−4.480	0.000
Part III	22.74	4.105	25.08	2.235	−3.089	0.003
Part IV	19.92	4.109	23.61	3.476	−4.220	0.000
LTP total score	86.71	10.905	99.63	7.684	−5.971	0.000

**Table 4:** Pearson's correlations between mother and father's PSI (Parental Stress Index) scores.

		PSI father PS	PSI father P-CDI	PSI father DC	PSI father stress tot
PSI mother PS	Pearson's correlation	.491^*∗∗*^	.272	.407^*∗*^	.455^*∗∗*^
	Sig. (2-tailed)	.001	.094	.010	.004
	*N*	39	39	39	39

PSI mother P-CDI	Pearson's correlation	.485^*∗∗*^	.381^*∗*^	.320^*∗*^	.452^*∗∗*^
	Sig. (2-tailed)	.002	.017	.047	.004
	*N*	39	39	39	39

PSI mother DC	Pearson's correlation	.481^*∗∗*^	.363^*∗*^	.576^*∗∗*^	.540^*∗∗*^
	Sig. (2-tailed)	.002	.023	.000	.000
	*N*	39	39	39	39

PSI mother stress tot	Pearson's correlation	.546^*∗∗*^	.374^*∗*^	.488^*∗∗*^	.540^*∗∗*^
	Sig. (2-tailed)	.000	.019	.002	.000
	*N*	39	39	39	39

^*∗*^ *Note* . PS: parental stress, CDI: parent-child dysfunctional interaction; DC: difficult child. ^*∗*^*p* < 0.05; ^*∗∗*^*p* < 0.01.

**Table 5:** Pearson's correlation between PSI and LTP scores.

		PSI mother P-CDI	PSI mother DC	PSI mother stress tot	PSI father P-CDI	PSI father DC	PSI father stress tot
LTP inclusion of partners	Pearson's correlation	−.358^*∗*^	−.089	−.271	−.159	−.109	−.132
	Sig. (2-tailed)	.025	.590	.095	.333	.511	.423
	*N*	39	39	39	39	39	39

LTP role implication	Pearson's correlation	.061	.160	.100	.327^*∗*^	.253	.326^*∗*^
	Sig. (2-tailed)	.713	.329	.545	.042	.120	.043
	*N*	39	39	39	39	39	39

LTP coconstruction	Pearson's correlation	−.382^*∗*^	−.097	−.238	.085	.148	.043
	Sig. (2-tailed)	.016	.557	.145	.605	.369	.793
	*N*	39	39	39	39	39	39

LTP support	Pearson's correlation	−.328^*∗*^	−.430^*∗*^	−.386^*∗*^	.012	−.168	−.117
	Sig. (2-tailed)	.041	.006	.015	.943	.308	.478
	*N*	39	39	39	39	39	39

LTP self-regulation	Pearson's correlation	−.072	−.042	−.053	−.373^*∗*^	−.249	−.299
	Sig. (2-tailed)	.664	.798	.750	.019	.126	.064
	*N*	39	39	39	39	39	39

LTP interactive mistakes during activities	Pearson's correlation	−.252	−.273	−.281	.143	.102	.046
	Sig. (2-tailed)	.121	.093	.083	.386	.535	.782
	*N*	39	39	39	39	39	39

LTP interactive mistakes during transitions	Pearson's correlation	−.015	−.053	.084	−.239	−.324^*∗*^	−.207
	Sig. (2-tailed)	.928	.747	.610	.142	.044	.207
	*N*	39	39	39	39	39	39

^*∗*^ *p* < 0.05.
